# Reciprocal regulation of rumen microbiota and epithelial genes in response to small peptide supplementation for feed efficiency in beef cattle

**DOI:** 10.3389/fvets.2025.1714827

**Published:** 2026-01-08

**Authors:** En Liu, Shujun Sun, Yawen Deng, Jiajia Liu, Jintao Xue, Mengmeng Li, Fuguang Xue

**Affiliations:** 1 School of Biology and Food Engineering, Fuyang Normal College, Fuyang, China; 2 Anhui Rural Revitalization Collaborative Technology Service Center, Fuyang, China; 3 School of Animal Science and Technology, Jiangxi Agricultural University, Nanchang, China; 4 Testing Center of Gaotang Market Supervision and Administration, Liaocheng, China

**Keywords:** small peptide, beef cattle, rumen nutrient digestibility, rumen microbiota, ruminal epithelial genes

## Abstract

**Introduction:**

Beef cattle during the finishing phase are predominately fed with high-cereal diets to promote rapid growth, which commonly caused surplus energy supply and nitrogen deficiency, disrupted rumen energy and nitrogen balance (RENB), and reduced feed efficiency. This study aims to determine the effects of small peptide (SP) supplement on reciprocal patterns between rumen microbiota and epithelial genes in regulating nutrient metabolism and feed efficiency of beef cattle.

**Methods:**

A total of sixty 12-month-old Simmenthal male beef cattle with the non-significant initial body weight were randomly assigned into the control treatment and arithmetically increased SP additional (0.2%, 0.4%, 0.6%, 0.8% and 1.0%) treatments. Each treatment contains 10 bulls with each bull was considered as one replicate. Growth performances, nutrient digestibility, rumen fermentable parameters, rumen microbiota, and rumen epithelial gene expressions were detected to determine the effects of SP on beef cattle.

**Results and discussion:**

0.6% and 0.8% of SP supplement showed the highest average daily weight gain (ADG), neutral detergent fiber (NDF) digestibility, and the lowest feed conversion ratio (FCR) among all treatments, which showed significant discrepancies compared with CON treatment (*P* < 0.05). Additionally, 0.6% of SP supplement treatment showed a significant higher content of acetate, and acetate/propionate ratio compared with 0.8% and CON treatments(*P* < 0.05). Therefore, 0.6% of SP supplement treatment was considered as the optimum supplement level and applied for further microbial and rumen epithelial gene expression analysis. SP supplement significantly increased the Alpha diversity and relative abundances of the *Acetitomaculum, Butyrivibrio, Pseudobutyrivibrio, Bifidobacterium,* and *Butyricicoccus* (*P* < 0.05), while decreased the *Saccharofermentans*, and *Selenomonas* (*P* < 0.05). Rumen epithelial results showed SP supplement up-regulated genes of *ATP10B, ACSF2, ADGRG6,* and *GALNT15,* while down-regulated genes of *ABCC3, GEM, PDK2,* and *ADIRF*. The differential expressed genes mainly enriched into the catalytic activity, pyruvate metabolism, metabolic pathways, protein digestion and absorption pathways. Conclusion. These findings demonstrate that SP supplementation enhances growth performance and rumen function and provide a viable nutritional strategy for improving feed efficiency in finishing beef cattle.

## Introduction

Beef cattle in the finishing phase are typically provided with cereal-rich diets to increase energy density, enhance feed efficiency, and accelerate growth rates. However, provision of concentrate-heavy diets requires an increased supply of rumen degradable nitrogen (RDN) to maintain the rumen energy and nitrogen balance (RENB), which is essential for supporting ruminal microbial proliferation and maximizing nutrient absorption (1–3). Even with the inclusion of protein-rich feeds, such as soybean meal, RDN content remains insufficient to match the high energy supply (4). RDN deficiency disrupts the homeostasis of the ruminal microbial ecosystem, impairs the synchronized utilization of carbon and nitrogen, reduces energy utilization efficiency, suppresses the proliferation of cellulolytic and amylolytic bacteria, and can induce metabolic disorders such as subacute ruminal acidosis (SARA) (3, 5). Therefore, high-RDN feedstuffs or additives are urgently needed to balance high-concentrate diets.

Non-protein nitrogen (NPN) supplementation has been considered a cost-effective approach to promote microbial protein (MCP) synthesis by stimulating the growth of urea-utilizing bacteria (6). However, a limited supplement quantity should be carefully administered to avoid ruminal metabolic disorder. Small peptides (SPs), derived from the enzymatic hydrolysis of crude proteins (CPs), can be directly assimilated by specific bacterial groups for microbial protein synthesis or can be further degraded into amino acids, which are subsequently converted into volatile fatty acids (VFAs), carbon dioxide (CO₂), and ammonia through deamination (7). Moreover, SPs provide a readily available nitrogen source for microbial proliferation, thereby potentially improving growth and production efficiency (8, 9).

Interestingly, previous studies have demonstrated that the addition of small peptides helps re-establish the RENB in the high-concentrate feeding process, significantly enhancing milk production in dairy cows and increasing daily weight gain in beef cattle (10–12). However, the underlying mechanisms linking SP supplementation to ruminal epithelial function and microbiota–epithelium crosstalk remain unclear. SP supplementation may enhance growth performance and carbohydrate degradation by modulating positive interactions between ruminal epithelial gene expression and cellulolytic/amylolytic bacteria. Therefore, this study enrolled 60 12-month-old Simmental male beef cattle to evaluate the effects of SP supplementation on growth performance and on the interactions between the rumen microbiota and epithelial gene expression.

## Materials and methods

### Animal preparation and experimental design

Animals were reared at Shandong Aoshida Animal Husbandry Development Co., Ltd., Gaotang, Shandong province. All care and experimental procedures followed the Chinese Guidelines for Animal Welfare and were approved by the Animal Care and Use Committee of Jiangxi Agricultural University (Approval number: JXAULL-20250218).

The small peptides (SP) used in this study were the same as those in our previous study (10, 11). Briefly, SPs were acquired through enzymatic hydrolysis of cottonseed protein combined with dephenolization and degraded into four fractions based on molecular weight (<1,000, 1,000–2000, 2000–5,000, and >5,000 Da), which accounted for 68.4, 16.7, 8.3, and 5.6% of the total peptides, respectively. The RDP proportion of SPs was calculated to be approximately 94.72% of the total protein content based on the following equation (13):


$$\mathrm{RDP}=A+B\frac{Kd}{Kd+\mathrm{Kp}}$$

where A represents non-protein nitrogen and soluble proteins, B represents potentially degradable proteins, Kd represents the rumen digestibility of B, and Kp represents the velocity of circulation in the rumen.

A total of 60 12-month-old Simmental male beef cattle with similar initial body weights (BWs) (336.4 ± 21.6 kg) were randomly divided into a control group and arithmetically increased SP added (from 0.2 to 1.0%) treatments for a 90-day-long feeding process (10–12). Each treatment contained 10 bulls, with each bull considered as a single replicate. The ingredients and nutritional levels of each treatment were formulated according to the feeding standard of Chinese beef cattle (NY-T-815-2004) (14) to meet or exceed the estimated nutritional requirements, and the details of the ingredients and nutrient composition of each treatment are shown in Table 1. Diets were fed twice daily at 06:00 and 18:00, and water was provided *ad libitum* throughout the trial.

**Table 1:** Ingredients and chemical composition of the fermented substrates (dry matter basis).

Items	CON	0.20%	0.40%	0.60%	0.80%	1.00%
Ingredients (%)						
Ground corn	30.2	30.3	30.4	30.5	30.6	30.7
Steam-flaked corn	12.5	12.5	12.5	12.5	12.5	12.5
Cottonseed meal	3.4	3.2	3	2.8	2.6	2.4
Soybean meal	12	11.8	11.6	11.4	11.2	11
Beet pulp	4.5	4.6	4.7	4.8	4.9	5
Chinese wildrye	10.2	10.2	10.2	10.2	10.2	10.2
Corn silage	20.5	20.5	20.5	20.5	20.5	20.5
DDGS^a^	3.1	3.1	3.1	3.1	3.1	3.1
Small peptide	0	0.2	0.4	0.6	0.8	1
NaCl	0.6	0.6	0.6	0.6	0.6	0.6
Premix^b^	3	3	3	3	3	3
Total	100	100	100	100	100	100
Chemical composition (%)						
DM	51.2	51.2	51.2	51.2	51.2	51.2
NE_mf_ (MJ/kg)^c^	7.26	7.26	7.26	7.26	7.26	7.26
EE	4.56	4.56	4.56	4.56	4.56	4.56
CP	17.1	17.1	17.1	17.1	17.1	17.1
RDP^d^	10.76	10.85	10.94	11.03	11.12	11.21
ADF	18.6	18.6	18.6	18.6	18.6	18.6
NDF	27.6	27.6	27.6	27.6	27.6	27.6
Starch	30.8	30.8	30.8	30.8	30.8	30.8
Ca	0.69	0.69	0.69	0.69	0.69	0.69
P	0.44	0.44	0.44	0.44	0.44	0.44

a DDGS, distillers dried grains with solubles.

b The components contained in the premix were as follows: Fe, 1,400 mg; Cu, 1,200 mg; Mn, 2,400 mg; Zn, 5,500 mg; Se, 40 mg; Co, 30 mg; I, 90 mg; VA, 900,000 IU; VD, 700,000 IU; and VE, 9,000 IU.

c NE_mf_ = {322LBW^0.75^ + [(2092 + 25.1 × LBW) × ADG/(1–0.3 × ADG)]} × F. Where, LBW, live body weight; ADG, average daily gain; F, correction coefficient.

d RDP was calculated using the equation: RDP = A + B[Kd/(Kd+Kp)]. Where, A = non-protein nitrogen and soluble proteins, B = potentially degradable proteins, Kd = rumen digestibility of B, and Kp = velocity of circulation in the rumen.

### Growth performances and apparent nutrient digestibility measurement

Body weight (BW) was recorded at both the beginning and end of the trial after a 12-h fasting period. Average daily gain (ADG), average daily feed intake (ADFI), and feed conversion ratio (FCR) were calculated based on daily records throughout the experimental period using the following equation.


$$\mathrm{FCR}=\frac{\text{average daily feed intake}\;(\mathrm{kg})}{\text{average daily weight gain}\;(\mathrm{kg})}$$

Feed and fecal samples were collected during the last 3 days to investigate nutrient digestibility. Briefly, the fecal samples from each bull were first mixed with 10% H_2_SO_4_ for nitrogen fixation. Dry matter (DM), crude protein (CP), ether extract (EE), calcium, and phosphorus in the feed and fecal samples were analyzed according to the Association of Analytical Communities (AOAC) (15). Neutral detergent fiber (NDF) and acid detergent fiber (ADF) were determined using the ANKOM A200i Fiber Analyzer (ANKOM Technology Co., New York, NY, United States).

### Rumen fermentation parameter measurement

Rumen fluid samples were collected 3 h after the morning feeding through esophageal tubing and divided into two portions on the final day of the trial. One portion was immediately analyzed for ruminal pH using a Testo 206-pH1 meter (Testo Instruments International (shanghai) CO., Shanghai, China), volatile fatty acids (VFAs) using a gas chromatograph (GC-2010, Shimadzu, Kyoto, Japan), ammonia-N (NH_3_-N) using a UV-2600 ultraviolet spectrophotometer (Tianmei Ltd., China) at the 700 nm wavelength, and microbial protein (MCP). The second portion of the rumen fluid was snap-frozen in liquid nitrogen and stored at −80 °C for subsequent microbiota analysis.

### Ruminal microbial community measurement

Ruminal microbial communities were analyzed following the methods described by Hall and Beiko (16). Briefly, microbial DNA was extracted and purified from the rumen fluid of the bulls in the CON and optimal SP groups using a Bacterial Genome DNA Extraction Kit (DP302, TIANGEN, TIANGEN BIOTECH (BEIJING) Co., Ltd) and a Qiagen Gel Extraction Kit (Qiagen, Hilden, Germany), respectively. DNA libraries were prepared using the TruSeq^®^ DNA PCR-Free Sample Preparation Kit (Illumina Inc., San Diego, United States) and sequenced on the Illumina HiSeq 4,000 platform (Illumina Inc., San Diego, United States). Raw tags were qualified under specific filtering conditions according to Quantitative Insights Into Microbial Ecology (QIIME, V2.0) (17), and sequences were clustered into operational taxonomic units (OTUs) at 97% similarity.

### Ruminal epithelial sampling and transcriptomic sequencing

Rumen endodermal epithelial samples were collected immediately after slaughter and placed in ice-cold phosphate-buffered saline (PBS). The tissues were washed five times with PBS containing 0.5 mg/mL amphotericin B and 100 μg/mL gentamicin, then rapidly frozen in liquid nitrogen. The transcriptomic sequencing method was used as described by Hrdlickova (18). Total RNA was extracted from each sample using an RNA kit (Takara, Takara Biomedical Technology (Beijing) Co., Ltd., Beijing, China) according to the manufacturer’s instructions and purified using Agencourt^®^ RNAClean™ XP (Beckman Coulter, Inc., Indianapolis, IN, United States). High-quality RNA was used to construct cDNA libraries for RNA sequencing (RNA-Seq) on an Illumina NovaSeq 6,000 platform (Illumina Inc., San Diego, CA). Differentially expressed genes (DEGs) were identified using DESeq2 (v1.42.0). Genes with an adjusted *p*-value (P_adj_) < 0.05 and an absolute fold change (|FC|) ≥ 2.0 were considered significantly differentially expressed and were further subjected to Gene Ontology (GO) and Kyoto Encyclopedia of Genes and Genomes (KEGG) enrichment analyses.

A total of eight DEGs, including four upregulated and four downregulated genes in the SP treatment, were selected for qRT-PCR verification analysis. Total RNA from all samples was first reverse-transcribed into cDNA using a Transcript First Strand cDNA Synthesis Kit (Bio-Rad Laboratories, Hercules, United States), and qPCR was performed using a Roche RT-PCR system (Roche, Applied Science, Mannheim, Germany). Gene-specific primers were designed with the Primer 5.0 software based on GenBank sequences (listed in Table 2). *GAPDH* was used as the reference gene.

**Table 2:** Primers of significantly differentially expressed genes.

Name	Primer	Sequence	Size
*GAPDH*	Forward	5′- GTCGGAGTGAACGGATTTGG-3’	178 bp
	Reverse	5′- CGTTCTCTGCCTTGACTGTG-3’	
*ABCC3*	Forward	5′- ACTCTACCCTGACACCACCT-3’	85 bp
	Reverse	5′- GCACATTGTTTGGGTCCACG-3’	
*GEM*	Forward	5′- AAGAGAACCCCTGGAACGTG-3’	606 bp
	Reverse	5′- GGGAATGTCCTCTGTCTGCC-3’	
*PDK2*	Forward	5′- ACTGCAACGTCTCTGAGGTG-3’	654 bp
	Reverse	5′- AGAAAGTTGGGTAGCTGACG-3’	
*ADIRF*	Forward	5′- CACAGATACCCCGAAGCCAT-3	204 bp
	Reverse	5′- AGAGGCCTGGTTAGCAGTCT-3’	
*ATP10B*	Forward	5′- ACTCCTCTCCACTGCCAAGA-3’	945 bp
	Reverse	5′- GAGAAGCCCTTCACGACACA-3’	
*ACSF2*	Forward	5′- TGTACCATTGCCTGGGTTCC-3’	937 bp
	Reverse	5′- TACAAGAAAGTTGGGCACAGA-3’	
*ADGRG6*	Forward	5′- TCACTCAGTGTGGGGATGTG-3’	260 bp
	Reverse	5′- ATAGGCCTTTGGCAGTTGCT-3’	
*GALNT15*	Forward	5′- ATTCTCCAGCTCAACCCAGC-3′	677 bp
	Reverse	5′- AGGCTCTGGCATTTTCCTCC-3’	

### Statistical analysis

Growth performance and rumen fermentation variables were first assessed for normality using the SAS proc. univariate procedure. Data were then analyzed using one-way ANOVA followed by the Student–Newman–Keuls (S-N-K) multiple comparison test (SAS Institute Inc., Cary, NC, United States). Significance was declared at a *p*-value of < 0.05.

Principle coordinate analysis (PCoA) that displayed the differential analysis on beta diversity of ruminal species complexity was applied using QIIME (Version 2.0) and displayed using the ggplot2 package in R software (Version 3.15.3, R Core Team, Vienna, Austria). Gene expression levels were normalized to GAPDH using the 2^−ΔΔCt^ method, and statistical differences were determined accordingly.

## Results

### Effect of SP supplementation on growth performance and apparent nutrient digestibility

The effects of SP supplementation on growth performance are presented in Table 3. Supplementation with 0.6 and 0.8% SP significantly decreased the FCR compared to the CON treatment (*p* < 0.05) and showed a tendency to increase ADG (*p* = 0.084). No other significant differences were observed in IBW, FBW, ADFI, and other indicators between the CON and SP supplementation treatments.

**Table 3:** Effects of small peptide supplementation on growth performance and nutrient digestibility in Simmental beef cattle (*n* = 10).

Items	Experimental treatments						SEM	*P*-value
	CON	0.20%	0.40%	0.60%	0.80%	1.00%		
IBW (kg)	335.8	337.2	334.7	336.9	331.8	335.2	14.16	0.683
FBW (kg)	609.4	614.4	620.9	639.3	634.2	631.3	18.27	0.104
ADFI (kg/d)	8.31	8.47	8.59	8.65	8.69	8.78	0.18	0.267
ADG (kg/d)	1.52	1.54	1.59	1.68	1.68	1.65	0.073	0.084
FCR	5.47^a^	5.50^a^	5.40^a^	5.15^b^	5.17^b^	5.34^a^	0.63	0.037
DM (%)	58.66	59.21	59.46	60.23	59.64	58.31	1.094	0.216
NDF (%)	64.24^b^	64.18^b^	64.34^b^	65.97^a^	65.85^a^	64.21^b^	0.541	0.043
ADF (%)	52.62	53.33	53.89	55.21	55.13	54.36	1.273	0.338
EE (%)	66.23	66.31	67.29	66.88	67.21	66.41	3.632	0.527
CP (%)	63.37	63.76	63.23	64.12	64.34	63.27	1.113	0.143
Ca (%)	44.09	44.12	44.78	44.96	45.21	45.06	2.371	0.356
P (%)	43.32	42.21	43.37	42.01	42.64	42.63	1.484	0.316

^a,b^ Means within a row with different letters differed significantly (*p* < 0.05); SEM, standard error of the mean; CON, control diet; DM, dry matter; NDF, neutral detergent fiber; ADF, acid detergent fiber; EE, ether extracts; CP, crude protein; Ca, calcium; P, phosphorus.

Regarding nutrient digestibility indicators, supplementation with 0.6 and 0.8% SP significantly increased the digestibility of NDF compared to other treatments (*p* < 0.05).

### Effect of SP supplementation on rumen fermentation parameters

Rumen fermentation parameters, including VFAs, rumen MCP, and NH_3_-N contents for each treatment, are shown in Table 4. Supplementation with 0.6, 0.8, and 1.0% SP significantly increased the MCP content (*p* < 0.05). In addition, 0.8 and 1.0% SP significantly increased the NH_3_-N content compared to the CON treatment (*p* < 0.05) but showed no significant difference from the 0.6% SP supplementation treatment. Furthermore, supplementation with 0.6% SP significantly increased acetate and butyrate contents while decreasing the propionate content compared to the CON group (*p* < 0.05). This shift causatively led to a significant increase in the A/P rate in the 0.6% SP group compared to the CON group (*p* < 0.05).

**Table 4:** Effects of small peptide supplementation on rumen fermentation parameters of Simmental beef cattle (*n* = 10).

Items	Experimental treatments						SEM	*P*-value
	CON	0.20%	0.40%	0.60%	0.80%	1.0%		
Ruminal pH	5.97	6.02	6.01	6.02	5.99	6.08	0.094	0.216
MCP (mg dL-1)	23.16^b^	23.69^b^	23.23^b^	25.34^a^	24.94^a^	24.57^a^	0.741	0.043
Acetate (mmol/L)	47.24^b^	47.85^b^	48.26^ab^	49.27^a^	47.28^b^	46.07^b^	1.273	0.038
Propionate (mmol/L)	17.49^a^	17.38^a^	16.11^b^	16.13^b^	16.75^b^	16.44^b^	0.632	0.027
Acetate/Propionate	2.70^b^	2.75^b^	3.00^a^	3.05^a^	2.82^b^	2.80^b^	0.113	0.043
Butyrate (mmol/L)	11.17^b^	12.12^b^	12.28 ^b^	13.75^a^	13.33^a^	13.01^ab^	0.879	0.036
Ammonia-N (mg/100 mL)	12.49^b^	13.41^ab^	13.54^ab^	13.77^ab^	13.96^a^	14.27^a^	0.981	0.006

^a,b^Means the different letters in the same row showed the significant discrepancy (*p* < 0.05); SEM, standard error of the mean; CON, control diet; MCP, microbial protein.

### Ruminal microbial community measurement

Based on the above parameters, the 0.6% SP supplementation treatment was selected as the optimal level for measuring ruminal microbial communities. A total of 18 phyla and 1,674 genera were identified across all samples after quality control, and all microbial communities are displayed in Supplementary Table S1. All data were used for *α*-diversity and *β*-diversity analyses.

#### α-diversity

The effects of SP supplementation on ruminal microbial α-diversity are shown in Table 5. Supplementation with SP significantly increased the Chao1, ACE, and Shannon indices compared to the CON group (*p* < 0.05), indicating increased species richness and diversity. No significant differences were detected in the Simpson index between the groups.

**Table 5:** Effects of small peptide supplementation on ruminal microbial α-diversity in Simmental beef cattle (*n* = 10).

Items	Experimental treatments		SE	*P-*value
	CON	SP		
Chao 1	2,324	2,548	47.43	0.003
Ace	2325.2	2492.6	38.04	0.021
Shannon	7.35	8.20	0.18	0.018
Simpson	0.97	0.98	0.015	0.175

SP, small peptide supplement treatment; CON, control treatment; SE, standard error.

#### β-diversity

**Figure 1:**
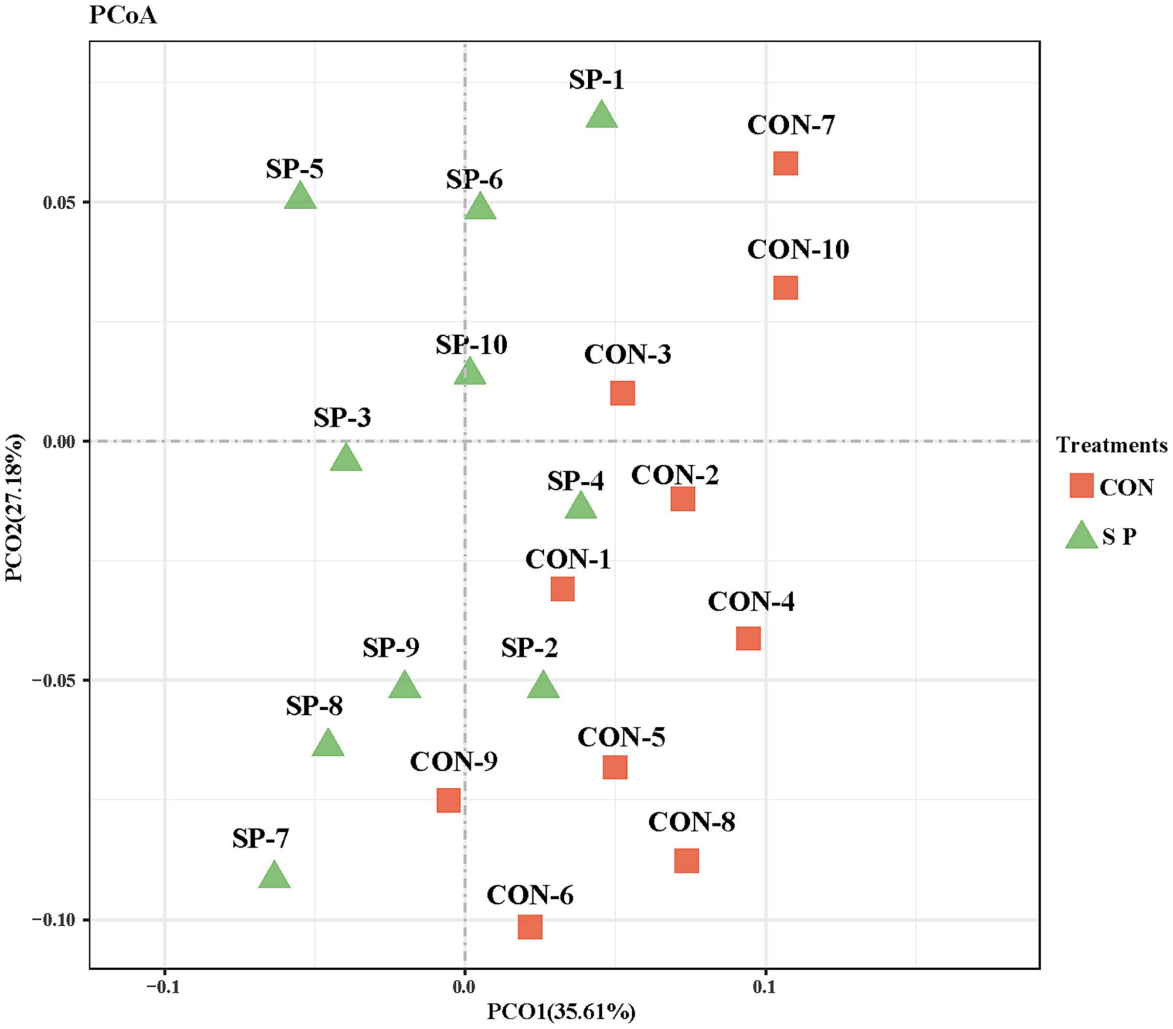
Principal coordinates analysis (PCoA) of rumen microbiota community structures between the small peptide supplement treatment and the control treatment. CON, control treatment; SP, small peptide supplement treatment.

PCoA revealed distinct differences in microbial community structure between the SP and CON groups (Figure 1). PCoA axes 1 and 2 accounted for 35.61 and 27.18% of the total alteration, respectively. Microbial communities in the SP group were clearly separated from those in the CON group through PCoA axes 1 and 2.

At the phylum level, as shown in Table 6, Bacteroidetes and Firmicutes were the two most abundant taxa. SP supplementation significantly increased the relative abundance of Bacteroidetes and Proteobacteria (*p* < 0.05) while significantly decreasing the abundance of Firmicutes (*p* < 0.05). At the genus level, as shown in Table 7, the relative abundance of *Acetitomaculum, Butyrivibrio, Pseudobutyrivibrio, Bifidobacterium*, and *Butyricicoccus* was significantly increased (*p* < 0.05), while the abundance of *Saccharofermentans* and *Selenomonas* was significantly decreased (*p* < 0.05) after SP supplementation. No other genera showed significant changes between the CON and SP groups.

**Table 6:** Effects of small peptide supplementation on the relative abundance of ruminal bacterial communities (%) at the phylum level (*n* = 10).

Items	Experimental treatments		SE	*P*-value
	CON	SP		
*Bacteroidetes*	33.51	36.78	1.274	0.041
*Firmicutes*	46.57	43.26	1.935	0.031
*Proteobacteria*	13.06	14.33	0.769	0.029
*Fibrobacteres*	1.95	1.64	0.058	0.228
*Spirochaetes*	0.95	1.04	0.166	0.187
*Actinobacteria*	1.42	1.25	0.114	0.108
*Verrucomicrobia*	0.32	0.34	0.031	0.071
*Cyanobacteria*	0.19	0.14	0.056	0.081
*Tenericutes*	0.14	0.16	0.017	0.301
Others	1.89	1.06	0.016	0.002

SP, small peptide supplement treatment; CON, control treatment; SE, standard error.

**Table 7:** Effects of small peptide supplementation in high-concentrate diets on the relative abundance of ruminal bacterial communities (%) at the genus level (*n* = 10).

Items	Experimental Treatments		SE	*P*-value
	CON	SP		
*g__Prevotella*	17.2	18.43	2.39	0.064
*g__Bacteroides*	16.7	14.84	1.09	0.105
*g__Succiniclasticum*	9.89	10.21	0.82	0.291
*g__Lachnospiraceae*	5.22	6.16	0.28	0.623
*g__Eubacterium*	4.84	4.97	0.21	0.141
*g__Rikenellaceae*	2.77	3.14	0.25	0.215
*g__Ruminococcus*	5.31	5.01	1.88	0.612
*g__Shuttleworthia*	1.61	1.34	0.52	0.532
*g__Prevotellaceae*	1.27	1.22	0.23	0.371
*g__Acetitomaculum*	0.73	1.28	0.33	0.023
*g__Lachnoclostridium*	0.66	0.73	0.22	0.995
*g__Saccharofermentans*	0.63	0.45	0.11	0.008
*g__Butyrivibrio*	0.48	0.58	0.05	0.048
*g__Ruminiclostridium*	0.21	0.28	0.08	0.503
*g__Lachnospira*	0.32	0.16	0.07	0.034
*g__Pseudobutyrivibrio*	0.11	0.18	0.03	0.009
*g__Acidaminococcus*	0.13	0.07	0.07	0.167
*g__Selenomonas*	0.11	0.04	0.04	0.001
*g__Lactobacillus*	0.05	0.06	0.05	0.936
*g__Pseudoramibacter*	0.04	0.02	0.03	0.089
*g__Bifidobacterium*	0.03	0.05	0.01	0.027
*g__Escherichia-Shigella*	0.01	0.01	0.003	0.681
*g__Succinivibrio*	0.03	0.01	0.003	0.001
*g__Streptococcus*	0.01	0.01	0.006	0.083
*g__Butyricicoccus*	0.01	0.03	0.007	0.009
Others	31.63	30.72	1.45	0.211

SP, small peptide supplement treatment; CON, control treatment; SE, standard error.

### Effects of SP supplementation on ruminal epithelial gene expression

**Figure 2:**
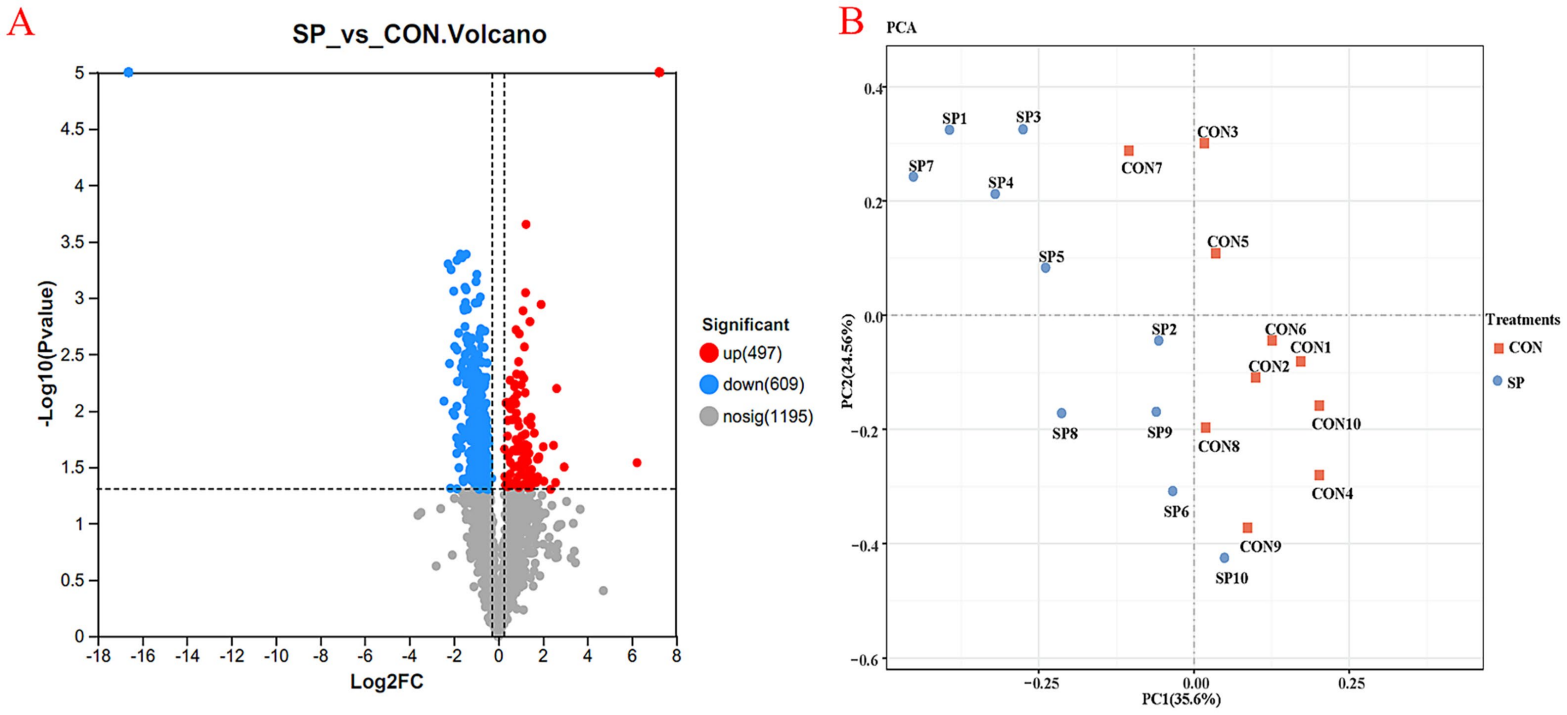
Differential analysis of the relative expression of ruminal epithelial genes between the small peptide supplement treatment and the control treatment. CON, control treatment; SP, small peptides supplement treatment. **(A)** Volcano plot showing differentially expressed ruminal epithelial genes between the CON and SP treatments. **(B)** Principal component analysis (PCA) depicting overall differences in ruminal epithelial genes between the CON and SP treatments.

A total of 11,370 mRNAs were identified across all samples after quality control, and all genes are displayed in Supplementary Table S2 and were used for differential analysis. As shown in Figure 2A, volcano plot analysis revealed 1,106 differentially expressed genes (DEGs), including 497 upregulated and 609 downregulated genes, in the SP group compared to the CON group. All identified genes are displayed in Supplementary Table S2. Principal component analysis (PCA) (Figure 2B) showed clear separation between the SP and CON groups, with PC1 and PC2 explaining 35.6 and 24.56% of the total variation, respectively.

**Figure 3:**
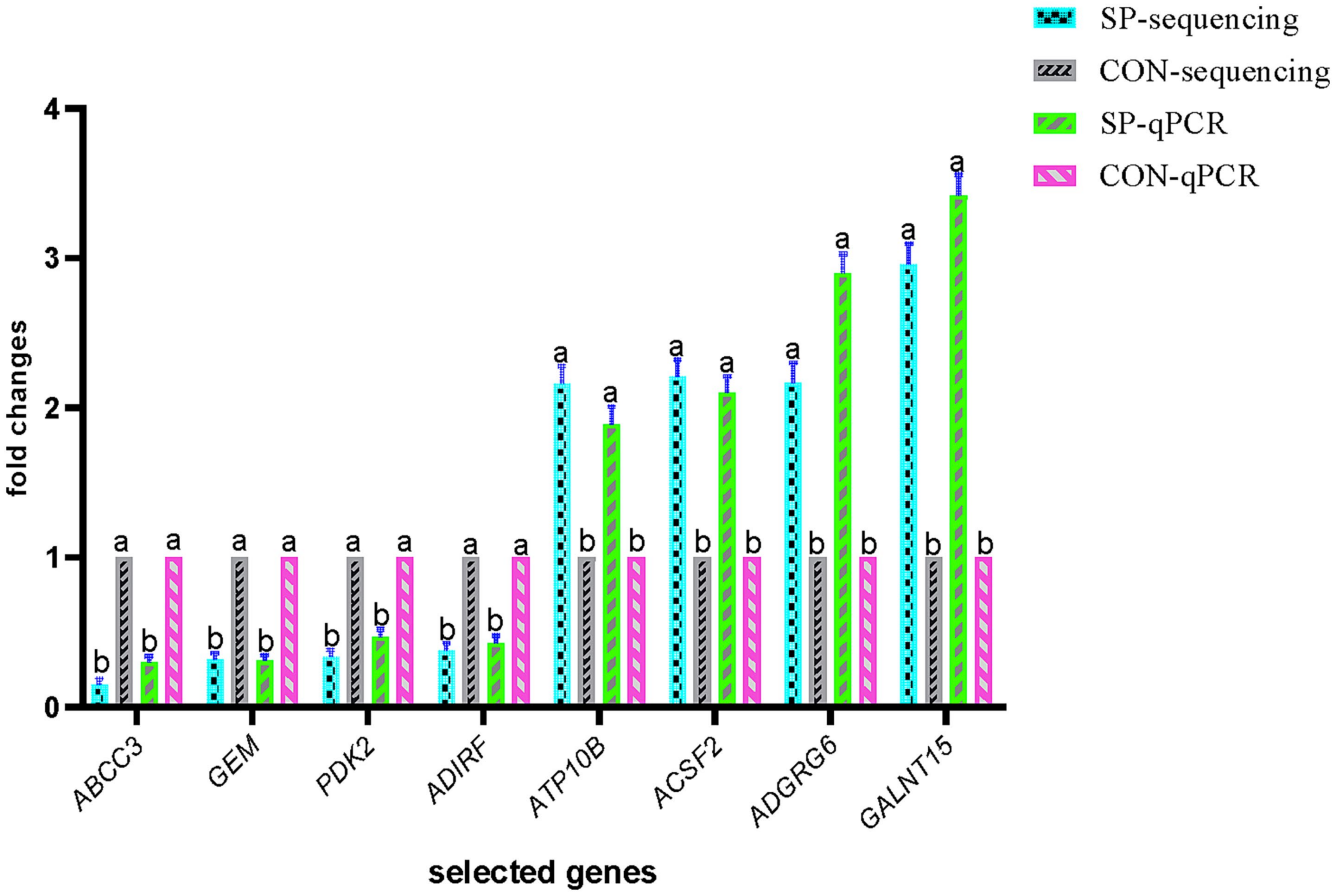
Identification and validation of differentially expressed ruminal epithelial genes between the small peptide supplement treatment and the control treatment. CON, control treatment; SP, small peptide supplement treatment.

Validation by qRT-PCR, as shown in Figure 3, further confirmed that SP supplementation significantly upregulated the expression of *ATP10B, ACSF2, ADGRG6,* and *GALNT15* while significantly downregulating the expression of *ABCC3, GEM, PDK2*, and *ADIRF* (*p* < 0.05).

**Figure 4:**
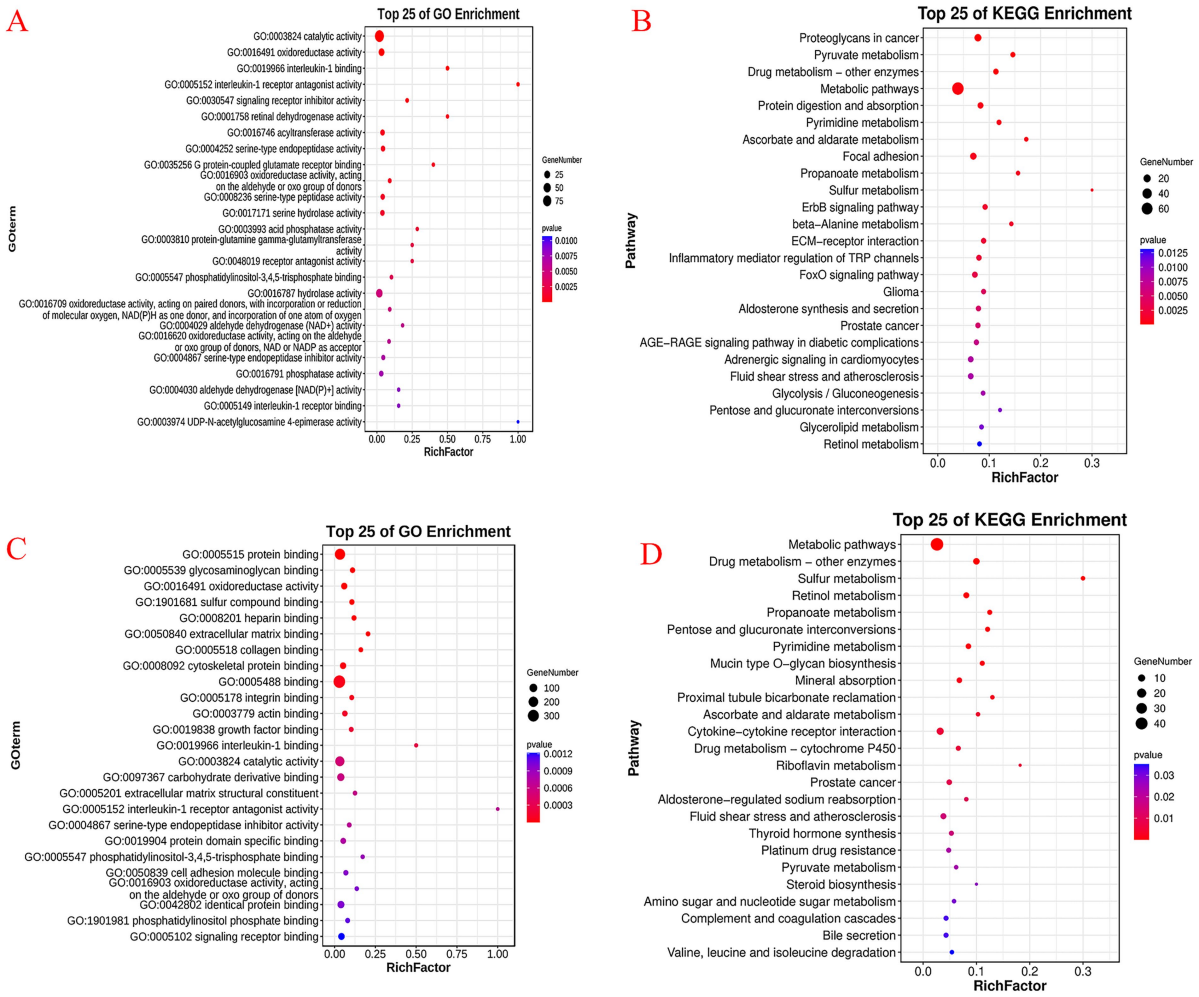
Gene ontology (GO) and pathway enrichment analyses of differentially expressed ruminal epithelial genes between the small peptide supplement treatment and the control treatment. CON, control treatment; SP, small peptides supplement treatment. **(A)** Gene ontology (GO) analysis of upregulated ruminal epithelial genes in the SP treatment compared to the CON treatment. **(B)** KEGG pathway enrichment analysis of upregulated ruminal epithelial genes in the SP treatment compared to the CON treatment. **(C)** Gene ontology (GO) analysis of downregulated ruminal epithelial genes in the SP treatment compared to the CON treatment. **(D)** KEGG pathway enrichment analysis of downregulated ruminal epithelial genes in the SP treatment compared to the CON treatment.

Functional enrichment analysis based on DEGs was conducted, and the results are shown in Figure 4. Figures 4A,B show that the significantly upregulated genes in the SP group were mainly enriched in catalytic activity and oxidoreductase activity and were primarily clustered into pyruvate metabolism, metabolic pathways, and protein digestion and absorption pathways. Figures 4C,D show that the significantly downregulated genes in the SP group were mainly enriched in the functions of protein binding, glycosaminoglycan binding, and oxidoreductase activity and were associated with metabolic pathways, sulfur metabolism, retinol metabolism, and propanoate metabolism.

### Regulatory effects of interactive crosstalk between ruminal epithelial genes and rumen microbiota on growth performance and rumen fermentation

**Figure 5:**
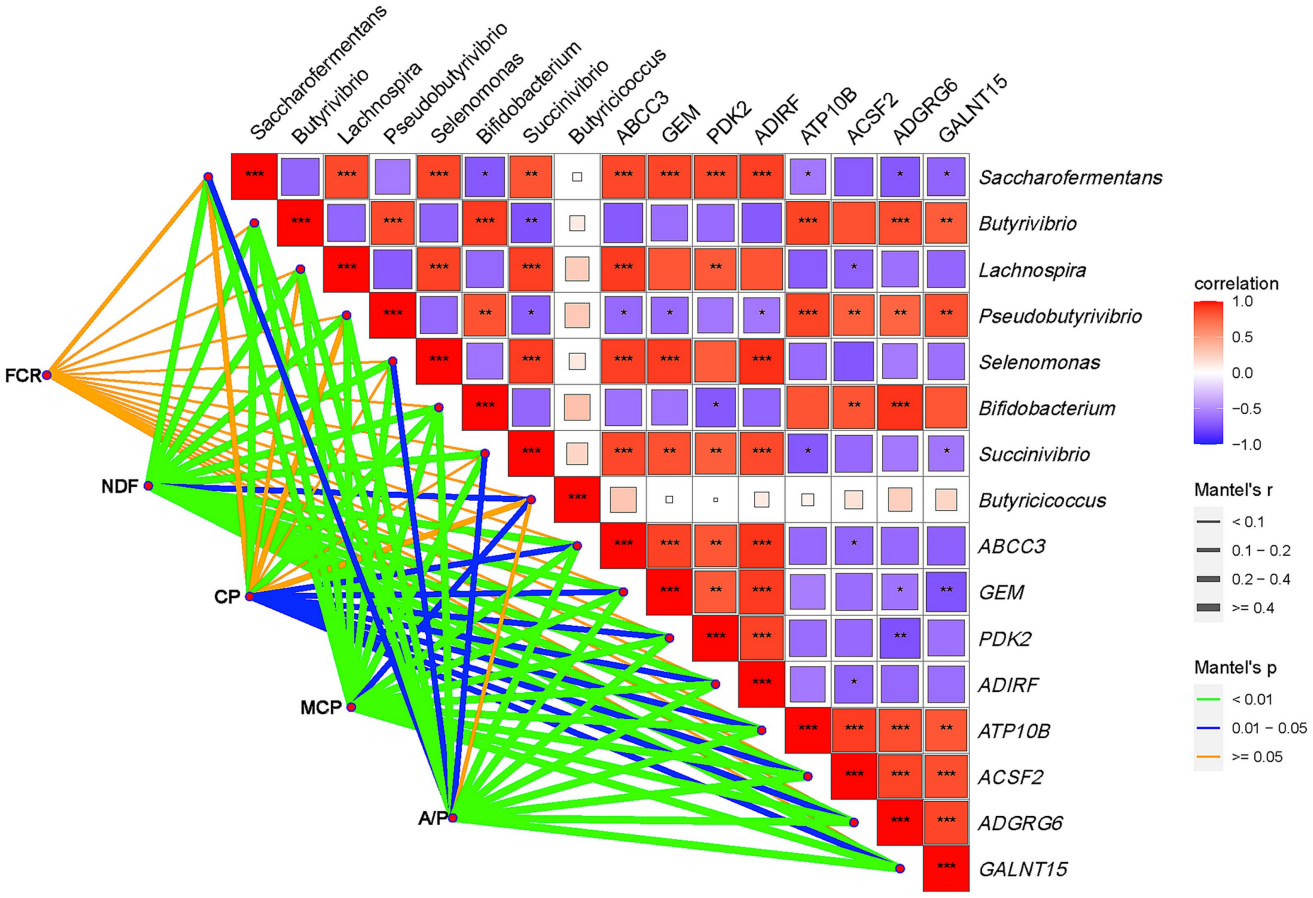
Interactive regulatory effects between rumen microbiota and ruminal epithelial genes on rumen fermentation, nutrient digestibility, and FCR. The red blocks represent positive correlations, while the blue blocks represent negative correlations. “*” Means a significant correlation (|r| > 0.55, *p* < 0.05), “**” means a significant correlation (|r| > 0.75, *p* < 0.01), and “***” means a significant correlation (|r| > 0.90, *p* < 0.001). The orange lines indicate no significant correlations. The blue lines indicate significant correlations (0.01 < *p* < 0.05). The green lines indicate highly significant correlations (*p* < 0.01). FCR, feed conversion ratio.

The correlation network between ruminal epithelial genes, rumen microbiota, and productive traits is shown in Figure 5. The selected genes and microbial communities showed weak correlations with the FCR but strong correlations with NDF, MCP, and the A/P ratio. CP degradability showed strong correlations with *Butyrivibrio* and *Bifidobacterium* but weaker correlations with other communities. All selected genes showed strong correlations with CP degradability. Specifically, *Saccharofermentans, Lachnospira, Selenomonas, and Succinivibrio* displayed positive correlations with the genes *ABCC3, GEM, PDK2,* and *ADIRF* and negative correlations with *ATP10B, ACSF2, ADGRG6, and GALNT15* in the regulation of CP and NDF degradation, MCP generation, and the ruminal A/P ratio. Conversely, microbial communities including *Butyrivibrio, Pseudobutyrivibrio, and Bifidobacterium* showed a completely inverse correlation with the above-mentioned genes compared to *Saccharofermentans, Lachnospira, Selenomonas, and Succinivibrio* in the regulation of CP and NDF degradation, MCP generation, and the ruminal A/P ratio.

## Discussion

Maintaining a stable RENB status plays a pivotal role in optimizing ruminant health and productivity, influencing key physiological processes such as microbial proliferation, nutrient degradation, epithelial development, and nutrient absorption (19). In the present study, the 0.6% SP supplement level in the high-concentrate diet feeding stage, compared to other supplement levels, showed a more effective enhancement of growth performance, mainly due to its contribution to restoring the RENB by improving the supply of RDP (1, 20). The beneficial effects of SP supplementation appear to operate through multiple interconnected mechanisms, which are discussed in detail below.

### Enhancement of ruminal microbial diversity and rumen fermentation

SP significantly elevated ruminal concentrations of acetate, butyrate, ammonia, and MCP, indicating an effective enhancement of rumen fermentation. Previous research has demonstrated that higher microbial diversity is generally associated with greater rumen fermentation (21). Consistent with the above-mentioned finding, our study showed significant increases in microbial *α*-diversity following SP supplementation, suggesting proliferated rumen microbiota and increased fermentation.

Under ruminal conditions, microbial communities proliferate autologously by relying on degradable nitrogen. SP supplementation provides a highly available nitrogen source for microbial proliferation, and when combined with the higher abundance of carbohydrates, it further stimulates microbial proliferation. This expansion of the microbial community inherently enhances fermentation activity, thereby increasing the production of VFAs and MCP (5, 22–24). Importantly, SP supplementation also addresses the RDP deficiency often present in high-concentrate diets, which likely helps re-establish the RENB. This shift notably stimulates cellulolytic bacteria such as *Acetitomaculum* and *Bifidobacterium* (25, 26), the key acetate- and butyrate-generating bacteria, which contributed to the improvement of acetate and butyrate content in the SP treatment compared with the CON group.

### Improvement of nutrient digestibility and absorption

Supplementation with 0.6% SP significantly decreased the FCR while increasing NDF digestibility, which played a critical role in indicating feed efficiency during the feeding process. Similar findings were reported by Zeng Yu (11) and EN Liu (12), suggesting enhanced feed efficiency and improved ruminal absorptive function. The underlying reasons may be attributed to the following aspects.

For beef cattle, nutrients degraded in the rumen are primarily absorbed across the ruminal epithelium for utilization in various physiological processes. This absorptive process is strongly influenced by the energy supply and the availability of butyrate. Previous studies have reported that butyrate plays a crucial role in stimulating ruminal epithelial growth and development (27, 28). Interestingly, the ruminal butyrate content significantly increased after SP supplementation, as shown in Table 4. The significantly altered microbial communities, as shown in Figure 2—especially the proliferation of predominant butyrate-producing genera *Butyrivibrio* and *Pseudobutyrivibrio*—may be the contributors to the increased butyrate levels. This, in turn, may further promote the assimilation of the ruminal epithelium.

In addition, nutrient transport across the ruminal epithelium is an energy-dependent process that requires acetate as a fundamental energy source, which is mainly generated by the end-product of rumen microbiota (29). The abundance of key acetate-generating bacteria, including *Acetitomaculum* and *Bifidobacterium,* significantly increased after SP supplementation, enhancing the acetate content and providing ample energy for nutritional transport. Moreover, SP supplementation upregulated ruminal epithelial genes such as *ATP10B* (30) and *ACSF2* (31), which are associated with cellular energy metabolism, thereby further facilitating nutrient absorption.

### Interactions between rumen microbiota and epithelial genes in response to SP supplementation in the regulation of nutrient metabolism

Our correlation analysis revealed a synergistic relationship between key ruminal microbial taxa and epithelial genes in regulating CP degradation and MCP synthesis, consistent with the findings of Lu (32) and Ge (33). This suggests a potential mechanism through which SP supplementation improves nitrogen utilization. These interactions can be discussed from three complementary perspectives.

#### SP supplementation increases rumen proteolytic microorganisms to promote CP degradation

Dietary protein undergoes complex microbial enzymatic catalysis to yield peptides, AAs, and ammonia, which are further utilized for microbial proliferation and MCP synthesis. Under ruminal conditions, proteolytic bacteria efficiently capture the dietary protein, which is further enzymatically converted into substrates that support MCP synthesis. Previous studies have shown that *Butyrivibrio* spp. possess strong proteolytic capacity (34, 35), which was significantly increased after SP supplementation in our study. The increased abundances of proteolytic bacteria may further enhance the proteolytic activity, degraded more crude proteins into peptides and AA acids, which further be metabolized into ammonia and therefore the ruminal ammonia concentration elevated.

#### SP supplementation increases ruminal nitrogen retention to promote MCP synthesis

Ruminal nitrogen metabolism is often inefficient due to rapid ammonia production exceeding MCP synthesis rates, leading to quantitative nitrogen losses. Slowing peptide breakdown helps reduce the conversion of protein to ammonia and increases ruminal nitrogen retention time (36, 37). SP Supplementation effectively complemented the ruminal peptide content and may have inhibited ammonia-producing enzymes by sending a deceleration signal. This process increased ruminal nitrogen retention time, facilitating more efficient activation of MCP-synthesizing enzymes and subsequently improving the MCP content.

#### Rumen microbiota and epithelial genes synergistically interact in energy-generation pathways to promote feed efficiency

MCP synthesis is an energy-intensive process that requires synergistic interactions between microbial communities and epithelial genes. SP supplementation significantly upregulated genes functionally linked to energy-generating pathways, including pyruvate metabolism (*ATP10B*) and protein digestion (*ACSF2*) (38, 39). The significantly increased epithelial genes synergistically interacted with the higher proliferated carbohydrate-degraded microbial communities, significantly enhanced microbial energy production and epithelial energy utilization. Collectively, these processes contributed to the observed improvements in feed efficiency.

## Conclusion

In conclusion, this research provides critical insights into optimizing high-concentrate beef finishing diets with small peptide (SP) supplementation, demonstrating that SP supplementation effectively enhances growth performance and rumen function by modulating ruminal microbial communities and epithelial gene expression. These findings suggest that SP supplementation offers a practical and efficient nutritional strategy to improve feed efficiency, nitrogen utilization, and metabolic stability in finishing beef cattle, ultimately contributing to sustainable beef production systems.

## Data Availability

The data presented in the study are deposited in the NCBI Sequence Read Archive (SRA, http://www.ncbi.nlm.nih.gov/Traces/sra/), accession number PRJNA753017.

## Supplementary material

SUPPLEMENTARY TABLE S1 16S rRNA results of all samples.

SUPPLEMENTARY TABLE S2 Identified gene expression results of rumen epithelium.
